# Differential diagnosis of Crohn’s disease and intestinal tuberculosis: development and assessment of a nomogram prediction model

**DOI:** 10.1186/s12876-022-02519-z

**Published:** 2022-11-16

**Authors:** Shaoxiong Zeng, Ying Lin, Jiaxiang Guo, Xi Chen, Qiong Liang, Xiaoming Zhai, Jin Tao

**Affiliations:** 1 Department of Gastroenterology, The Third Affiliated Hospital of Sun Yat-Sen University, No. 600 Tianhe Road, 510630 Guangzhou, Guangdong Province China; 2 Department of Radiology, The Third Affiliated Hospital of Sun Yat-Sen University, No. 600 Tianhe Road, 510630 Guangzhou, Guangdong Province China; 3 Department of Pathology, The Third Affiliated Hospital of Sun Yat-Sen University, No. 600 Tianhe Road, 510630 Guangzhou, Guangdong Province China

**Keywords:** Crohn’s disease, Intestinal tuberculosis, Differential diagnosis, Prediction model, Nomogram

## Abstract

**Background:**

China is a region with a high incidence of tuberculosis, and the incidence of IBD has also been rising rapidly in recent years. Differentiating Crohn’s disease(CD) from intestinal tuberculosis (ITB) has become a very challenging issue. We aimed to develop and assess a diagnostic nomogram to differentiate between CD and ITB to improve the accuracy and practicability of the model.

**Methods:**

A total of 133 patients (CD 90 and ITB 43) were analyzed retrospectively. Univariate and multivariate logistic regression analysis was included to determine the independent predictive factors and establish the regression equation. On this basis, the nomogram prediction model was constructed. The discrimination, calibration and clinical efficiency of the nomogram were assessed using area under the curve(AUC), C-index, calibration curve, decision curve analysis (DCA) and clinical impact curve.

**Results:**

T-SPOT positive, cobblestone appearance, comb sign and granuloma were significant predictors in differentiating CD from ITB. Base on the above independent predictors, a diagnostic nomogram was successfully established. The sensitivity, specificity, accuracy of the prediction model are 94.4%, 93.0%, 94.0% respectively. The AUC and the C-index of the prediction model are both 0.988, which suggest that the model had a good discrimination power. The calibration curve indicated a high calibration degree of the prediction model. The DCA and clinical impact curve indicated a good clinical efficiency of the prediction model which could bring clinical benefits.

**Conclusion:**

A nomogram prediction model for distinguishing CD from ITB was developed and assessed, with high discrimination, calibration and clinical efficiency. It can be used as an accurate and convenient diagnostic tool to distinguish CD from ITB, facilitating clinical decision-making.

**Supplementary Information:**

The online version contains supplementary material available at 10.1186/s12876-022-02519-z.

## Introduction

Crohn’s disease(CD) and intestinal tuberculosis (ITB) are both chronic granulomatous diseases of the intestine that share similar clinical manifestations, endoscopic findings, computed tomographic enterography (CTE) and histological features [1, 2]. The natural history and prognosis of CD and ITB are distinct. If early diagnosis and treatment are proper, intestinal tuberculosis can be completely cured. On the contrary, CD is an incurable lifelong disease with alternating recurrence and remission, requiring long-term maintenance treatment. As for therapeutic strategies, if ITB is treated with immunosuppressive and biological agents after being misdiagnosed as CD, it may lead to tuberculosis spread infection and even aggravate patient’s condition. Likewise, if CD patients misdiagnosed as ITB, or when it is difficult to distinguish, empirical anti-tuberculosis therapy will delay the diagnosis and treatment of CD, leading to the aggravation of CD patient’s condition, such as intestinal perforation, fistulas and other serious complications [2]. Misdiagnosis or delayed diagnosis will not only affect the effect of treatment but also increase the medical burden of patients and society [3]. Therefore, it is very important to make a correct differential diagnosis and take reasonable treatment measures in the early stage.

The “gold standard” for the diagnosis of intestinal tuberculosis, including typical granulomas with caseous necrosis, positive staining of acid-fast bacilli or positive culture of Mycobacterium tuberculosis, has high diagnostic value. However, the positive rate of these gold standard is low, which limits its value in clinical application. According to the literature, the positive rate of granulomas with caseous necrosis, acid-fast bacilli staining and Mycobacterium tuberculosis culture is only about 11.1%, 17.3% and 29.3% respectively [4]. Serological testing anti-saccharomyces cerevisiae antibody (ASCA) is considered as a serological marker of CD and widely used in diagnosis and differential diagnosis of inflammatory bowel disease [5]. However, the role of ASCA in distinguishing CD from ITB is controversial. It has been reported that serum tests such as ASCA can not distinguish Crohn’s disease from intestinal tuberculosis [6]. In addition, it was reported that the rate of misdiagnosis between these 2 diseases reaches 50–70% [7]. Epidemiological surveys in the Asia-Pacific region show that the incidence of inflammatory bowel disease is on the rise in Asia, with a regional incidence of 1.37 per 100,000 population per year in Asia.China has one of the highest incidence of IBD in Asia at 3.44 per 100,000 population per year, with the incidence of CD at 1.22 per 100,000 population per year [8]. According to the World Health Organization (WHO) report, China ranks second in the number of people with tuberculosis, with an incidence rate of 63.07 per 100,000 population per year, second only to India, indicating that China is still a high burden country for tuberculosis [9].Therefore, in China, where tuberculosis is prevalent and the incidence of CD is increasing, the differential diagnosis of these two diseases has become a very challenging problem, which is worthy of attention.

Conventionally, CD and ITB are distinguished based on clinical and ileocolonoscopic features, which have great limitations due to lack of diagnostic accuracy. Therefore, if we can integrate the predictive value indicators of clinical manifestations, endoscopic findings, CTE and histological features of these two diseases, and establish a diagnostic prediction model to distinguish CD from ITB, it is expected to obtain a diagnostic method with high accuracy and clinical maneuverability. Previously, a series of studies on establishing prediction model have been carried out, but these studies have limitations such as small sample size, retrospective study, including only parts of examination methods, complex formulas, inconvenience in clinical use and lack of clinical utility [10–13]. Nomograms are widely used as prognostic devices in oncology and medicine based on multivariate logistic regression. With the ability to generate an individual probability of a clinical event by integrating diverse prognostic and determinant variables, nomograms meet our desire for biologically and clinically integrated models with the great value in clinical practice [14].

Thus, the purpose of this study is to explore the valuable diagnostic indices in clinical manifestations, endoscopic findings, CTE and histological features between CD and ITB. On this basis, develop and assess a nomogram prediction model to differentiate between CD and ITB by synthesizing the valuable diagnostic indices, so as to improve the accuracy and practicability of the prediction model.

## Methods

### Patients

Patients with a definite diagnosis of CD or ITB were recruited for the study from the department of gastroenterology, the Third Affiliated Hospital of Sun Yat-sen University during the period from January 2011 to December 2020. Exclusion criteria were:(i) previous history of intestinal resection;(ii)definitive treatment including anti-TB drugs, immunosuppressive drugs, and chemotherapeutic agents before their referral to the hospital; (iii)being younger than 15 years; (iv) The clinical data including clinical manifestations, laboratory examination, endoscopic appearances, radiological and histopathological features are incomplete due to various reasons;(v)diagnosis was uncertain or was changed from Crohn’s disease or ITB to another disease during follow-up.

### Diagnostic criteria for CD and ITB

The diagnosis of CD was conformed to the European Crohn’s and Colitis Organization guidelines, a combination of clinical evaluation,endoscopic, histological, and radiological features and/or biochemical investigations [15]. All patients with CD had been followed up for at least 1 year in our clinical center.

The diagnosis of Intestinal tuberculosis was based on at least one of the following criteria is met:(i) demonstration of caseating granuloma on histological investigation; (ii) positive staining for acid-fast bacilli on smear or on histological; (iii) positive culture for acid-fast bacilli on tissue; (iv) Strong suspicion of tuberculosis by both clinical and histological characteristics,together with a sensitive response to antituberculous treatment; (v)complete clinical recovery with endoscopic mucosal healing after 6 months or longer standard antituberculosis treatment and no recurrence 9–12 months after the initiation of antituberculosis therapy in the follow-up [10, 16].

### Data collection

Detailed data of the patients regarding demographics (age, sex, history of smoking), clinical manifestations (abdominal pain, diarrhea, bloody stool, constipation, fever, night sweats, ascites, weight loss, abdominal mass, intestinal obstruction, perianal lesions, extra-intestinal manifestations and pulmonary tuberculosis), laboratory examination (T cell spot test), endoscopic findings (longitudinal ulcers, transverse ulcers, aphthous ulcers, cobblestone appearance, skip lesions, patulous ileocecal valve, intestinal stricture, mucosal bridge, scars or pseudopolyps, and location of lesion involvement), CTE features ( morphology of involved bowel segments including concentric thickening, asymmetrical thickening, skip lesions, segmental small-bowel involvement; type of enhancement pattern including target sign and homogeneous enhancement; mesenteric changes including comb sign and mesenteric fibrofatty proliferation; features of lymph nodes including central necrosis, calcification, greater than 1 cm, inhomogeneous enhancement or homogeneous enhancement; Others including peritoneal thickening, ascites and fistula and abscess), pathological features(chronic active inflammation; abnormal crypt structure including crypt branching, budding, distortion, atrophy;cryptitis; crypt abscess; granuloma; transmural inflammation; fissure-like ulcers and caseous necrosis) were documented in all patients. The CTE images were independently analyzed by experienced abdominal radiologists who were blinded to clinical, endoscopic, and laboratory data. The biopsies were then examined by pathologists with special interest in gastroenterology blinded about the clinical data and final diagnoses of the patients.

### Study design

The flow chart of this study is shown in Supplementary Fig. 1. The study was constructed in 3 steps as follows: step 1, univariate logistic regression analyses were conducted to determine statistically significant parameters potentially important in differentiating CD from ITB; step 2, parameters with statistical significance (*P*<0.05) were further analyzed by multivariable logistic regression (LR method) to further determine the independent predictive factors and establish a predictive model, displayed as nomogram to provide clinician with an intuitive and quantitative tool to predict the probability of CD; step 3, evaluation and validation of the nomogram prediction model. The study was approved by the Institutional Ethics Committee of Third Affiliated Hospital of Sun Yat-Sen University (registration number: 02-125-01).

### Statistical analysis

Statistical software SPSS 25.0 was used for statistical analysis. The t tests were used to compare mean values of appropriate parameters in the two groups. Chi-squared test or Fisher’s exact probability test with odds ratio was used to test the significance of differences in frequency of the various parameters between CD and ITB. In addition, parameters with statistical significance *(P*<0.05) were further analyzed by multivariable logistic regression (LR method) to build a predictive model. The nomogram is constructed by R software 4.0.2. The discrimination performance of the nomogram prediction model was evaluated by the area under the receiver-operating characteristic curve (AUROC) and the consistency index (C-index). Calibration curves were plotted to assess the calibration of the nomogram prediction model. Decision curve analysis (DCA) and clinical impact curve was conducted to determine the clinical usefulness of the nomogram. DCA compared the net benefits of each prediction model at any threshold probability. The net benefit was calculated by subtracting the proportion of all patients who are false positive from the proportion of the patients who are true positive and by weighing the relative harm of forgoing interventions compared with the negative consequences of an unnecessary intervention. The nomogram was subjected to bootstrapping validation (1,000 bootstrap resamples) to calculate a relatively corrected C-index. The reported statistical significance levels were all 2-sided, with statistical significance set at 0.05.

## Results

### Demographic features of patients with CD and ITB

A total of 150 patients were screened for the development of the prediction model. Of those, 17 patients were excluded from analysis: 1 patient with previous history of intestinal resection and 16 patients with inadequate data collection. Therefore, the remaining 133 patients (90 patients with CD and 43 with ITB) were finally enrolled and analyzed for the development group. The demographic features of patients with CD and ITB are summarized in Supplementary Table 1. The male-to-female ratio was 69 : 21 in CD and 29 : 14 in ITB (*P* = 0.258). The mean ages ± standard deviation (SD) of patients with CD and ITB, respectively, were 31.2 ± 11.8 and 42.7 ± 14.8 (*P <* 0.001). There was no statistically significant difference in gender ratio between the two groups. The age of ITB was statistically significantly older than that of CD and the proportion of ITB patients with a smoking history was statistically significantly higher than those of CD patients.

**Table 1:** Independent predictors in differentiating CD and ITB in multivariate logical regression analysis

Variable	β	*P*	OR	95% CI (lower)	95% CI (upper)
T-SPOT positive	-5.457	0.000	0.004	0.000	0.054
Cobblestone appearance	3.353	0.041	28.589	1.155	707.746
Comb sign	4.436	0.003	84.397	4.461	1596.677
Granuloma	-2.967	0.039	0.051	0.003	0.859
Constant	0.340	0.821	1.405		

### Univariate analysis for differentiation of CD and intestinal tuberculosis

#### Clinical and laboratory features

The main presenting symptoms in both CD and ITB patients were recurrent abdominal pain, diarrhea and weight loss. Diarrhea (CD:64.4% vs. ITB:44.2%, *P* = 0.027) and perianal disease (CD:15.6% vs. ITB:0.0%, *P* = 0.015) were significantly more common in patients with CD than in patients with ITB, while ascites (CD:0.0% vs. ITB:9.3%, *P* = 0.010), pulmonary tuberculosis (CD:0.0% vs. ITB:60.5%, *P* < 0.001) and T cell spot test (T-SPOT) positive (CD:7.8% vs. ITB:93.0%, *P* < 0.001) were found more often in patients with ITB than in those with CD. There were no significant difference between the two groups in abdominal pain, hematochezia, constipation, fever, night sweats, weight loss, abdominal mass, intestinal obstruction, and extraintestinal manifestations (Supplementary Table 2).

### Endoscopic features: types of lesions

The endoscopic findings of longitudinal ulcer (CD: 38.9% vs. ITB: 2.3%, *P* < 0.001), skip lesions(CD: 63.3% vs. ITB: 14.0%, *P* < 0.001), cobble stone appearance (CD: 31.1% vs. ITB: 4.7%, *P* = 0.001) were significantly more common in patients with CD than in patients with ITB. However, the endoscopic findings of transverse (ring-shaped) ulcers (CD: 4.4% vs. ITB: 67.4%, *P* < 0.001) were significantly more common in patients with ITB than in patients with CD. There was no statistical difference in endoscopic findings of aphthous ulcer, patulous ileocecal valve, scars or pseudopolyps, luminal stricture and mucosal bridge (Supplementary Table 3)(Supplementary Fig. 2).

### Endoscopic features: area of involvement

Intestinal ulcers are more common in the right colon in both Crohn’s disease and intestinal tuberculosis, especially in the ileocecum. Involvement in the terminal ileum (CD: 72.2% vs. ITB: 51.2%, *P* = 0.017), transverse colon(CD: 44.4% vs. ITB: 23.3%, *P* = 0.018), descending colon(CD: 46.7% vs. ITB: 16.3%, *P* = 0.001), sigmoid colon (CD: 50.0% vs. ITB: 14.0%, *P* < 0.001) and the rectum (CD: 40.0% vs. ITB: 7.0%, *P* < 0.001) was significantly more frequent in patients with CD than ITB. There was no significant difference in the involvement of the ileocecal valve, cecum and ascending colon in patients with CD and ITB (Supplementary Table 3).

### Radiological features

The CTE features in the two groups are summarized in Supplementary Table 4. Seven parameters, namely asymmetrical bowel wall thickening, skip lesion, segmental small-bowel lesions, target sign, the comb sign, mesentery fibrofatty proliferation and homogeneous enhancement of lymph nodes were significantly more common in Crohn’s disease patients. One parameter, namely homogeneous enhancement of bowel wall was more frequently observed in patients with ITB (Supplementary Fig. 3).

### Histological features

The histological features of patients with CD and ITB are summarized in Supplementary Table 5. Granulomas were found in 37.2% of patients with ITB and in 16.7% of patients with CD (*P* = 0.009) with significant statistical difference. Caseous necrosis was seen in 7.0% of patients with ITB but in none of the patients with CD (*P* = 0.032). Moreover, the histological findings of crypt abscesses and crypt atrophy were not significantly different between the groups (Supplementary Fig. 4).

### Results of multivariate logistic regression with binary outcome (CD vs. ITB)

On multivariate analysis, T-SPOT positive (OR 0.004, 95%CI 0.000–0.054), cobblestone appearance ( OR 28.589, 95%CI 1.155–707.746), comb sign (OR 84.397, 95%CI 4.461–1596.677) and granuloma (OR 0.051, 95% CI 0.003–0.859) were found to be significant predictors in differentiating CD and ITB (Table 1).

### Nomogram construction

**Fig. 1:**
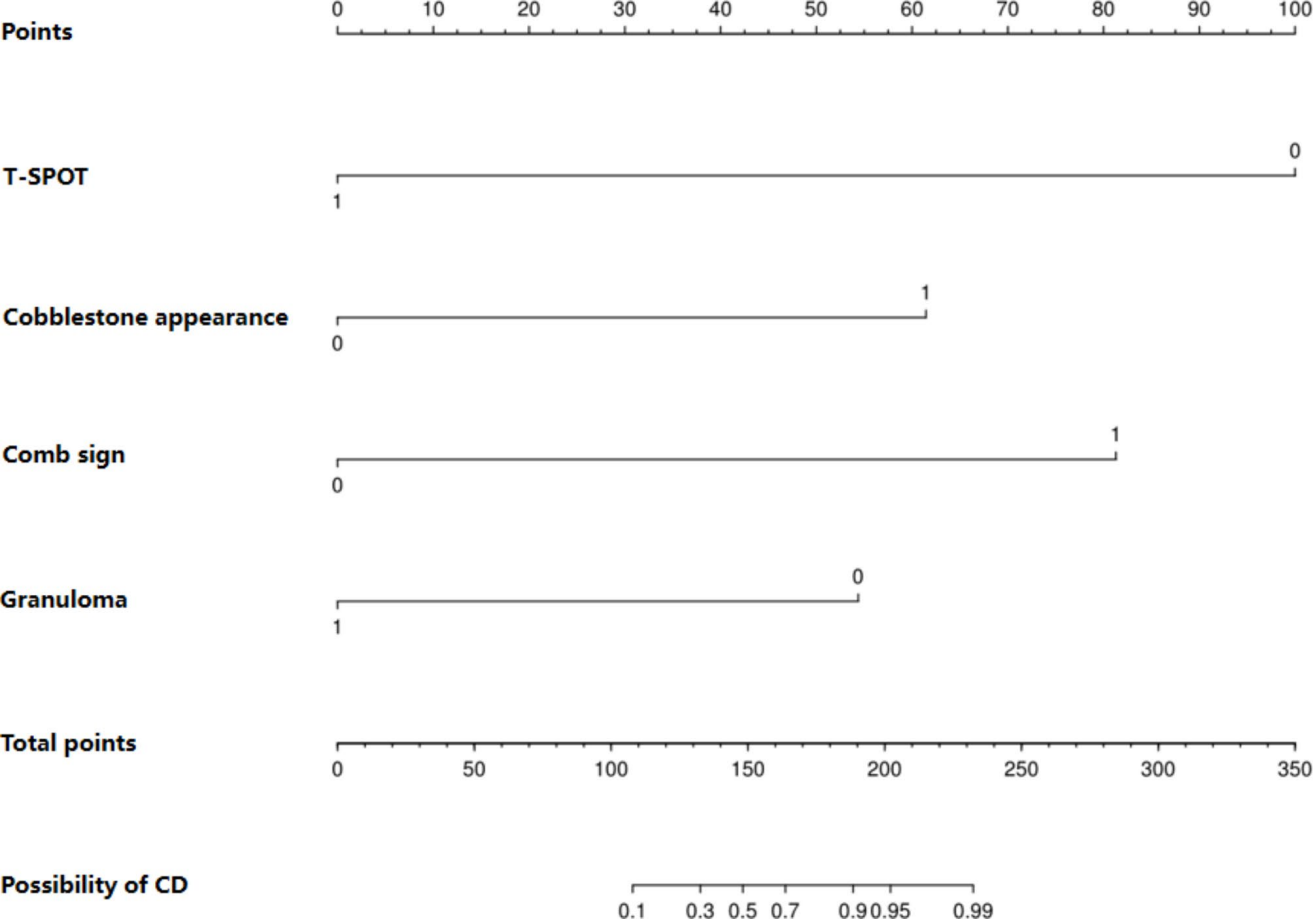
Nomogram prediction model to differentiate between Crohn’s disease and Intestinal tuberculosis

The diagnostic equation built for model was LogitP = 0.340–5.457 * T-SPOT positive + 3.353 * cobblestone appearance + 4.436 * comb sign − 2.967 * granuloma based on the basis of multivariable logistic regression, and the result was displayed as nomogram shown in Fig. 1. In the nomogram, each variable has a corresponding score according to the value, which was read out by drawing a line straight upward from each predictor to the point axis, and after calculating the total scores of the 4 variables, the possibility of diagnosing a patient as having CD was intuitively demonstrated.

### Assessment the performance of nomogram

**Fig. 2:**
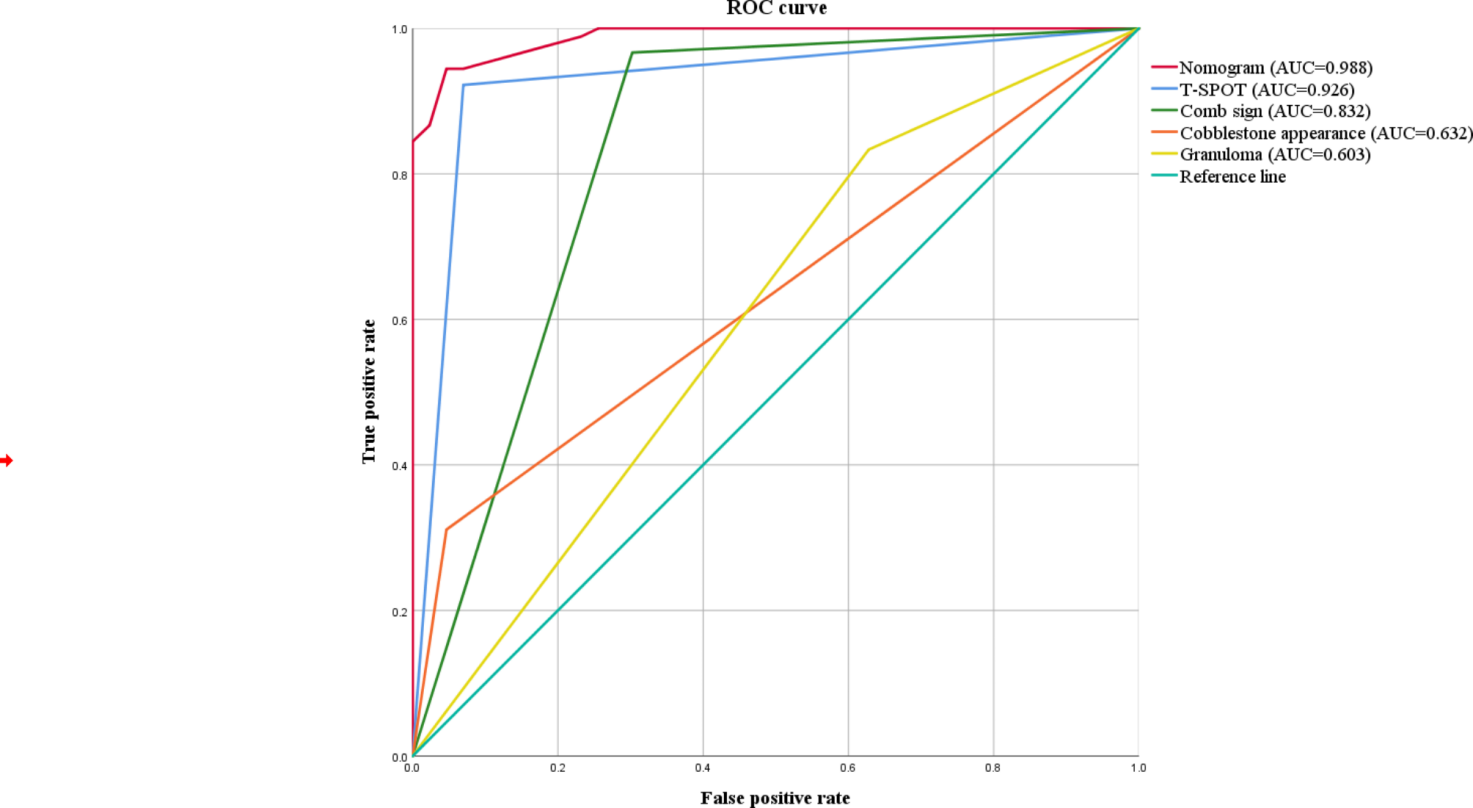
The ROC curve and the area under the curve of the nomogram prediction model and independent predictive factors

**Fig. 3:**
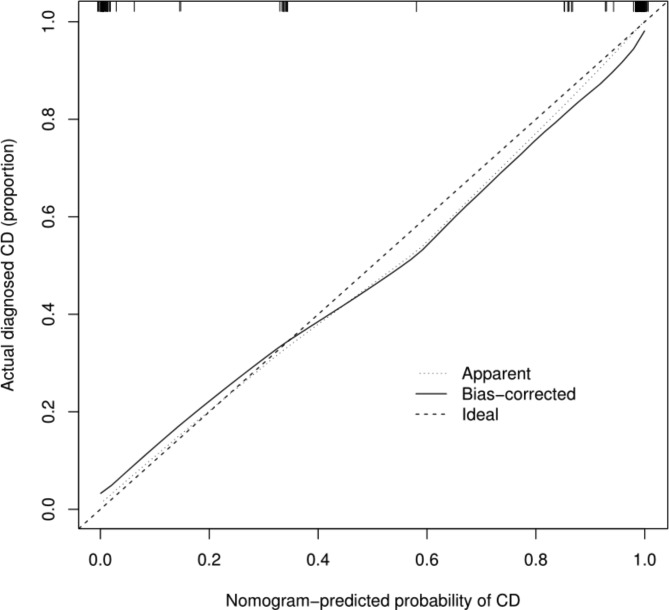
The calibration curve of the nomogram prediction model

The ROC curve of the nomogram prediction model was analyzed to evaluate the model’s diagnostic effect. The area under the curve of model was 0.988, which was superior to 0.926 in T-SPOT positive, 0.832 in comb sign, 0.632 in cobblestone appearance and 0.603 in granuloma, suggesting that the nomogram was superior to predict the probability of CD (Fig. 2). The sensitivity, specificity, accuracy, positive predictive value and negative predictive value of the nomogram prediction model were 94.4%, 93.0%, 94.0%, 96.6% and 88.9% respectively in differentiating CD from ITB(Table 2). The C-index for the prediction nomogram was 0.988 for the cohort, and was confirmed to be 0.981 through bootstrapping validation, which suggested the model’s good discrimination. The calibration curve was generated in this study, which showed that the apparent line and a bias-corrected line only slightly deviated from the ideal line, indicating a good consistency and a high degree of calibration(Fig. 3). Collectively, these findings show that the nomogram prediction model constructed with the above four indicators had an accurate predictive value for differential diagnosis of Crohn’s disease and intestinal tuberculosis.

**Table 2:** Independent predictors in differentiating CD and ITB in multivariate logical regression analysis

	AUC	sensitivity	specificity	accuracy	PPV	NPV
Nomogram	0.988	0.944	0.930	0.940	0.966	0.889
T-SPOT positive	0.926	0.922	0.930	0.925	0.965	0.851
Comb sign	0.832	0.967	0.698	0.880	0.870	0.909
Cobblestone appearance	0.632	0.311	0.953	0.519	0.933	0.398
Granuloma	0.603	0.833	0.372	0.684	0.735	0.516

PPV, positive prediction value; NPV, negative prediction value

### Clinical use of the nomogram

**Fig. 4:**
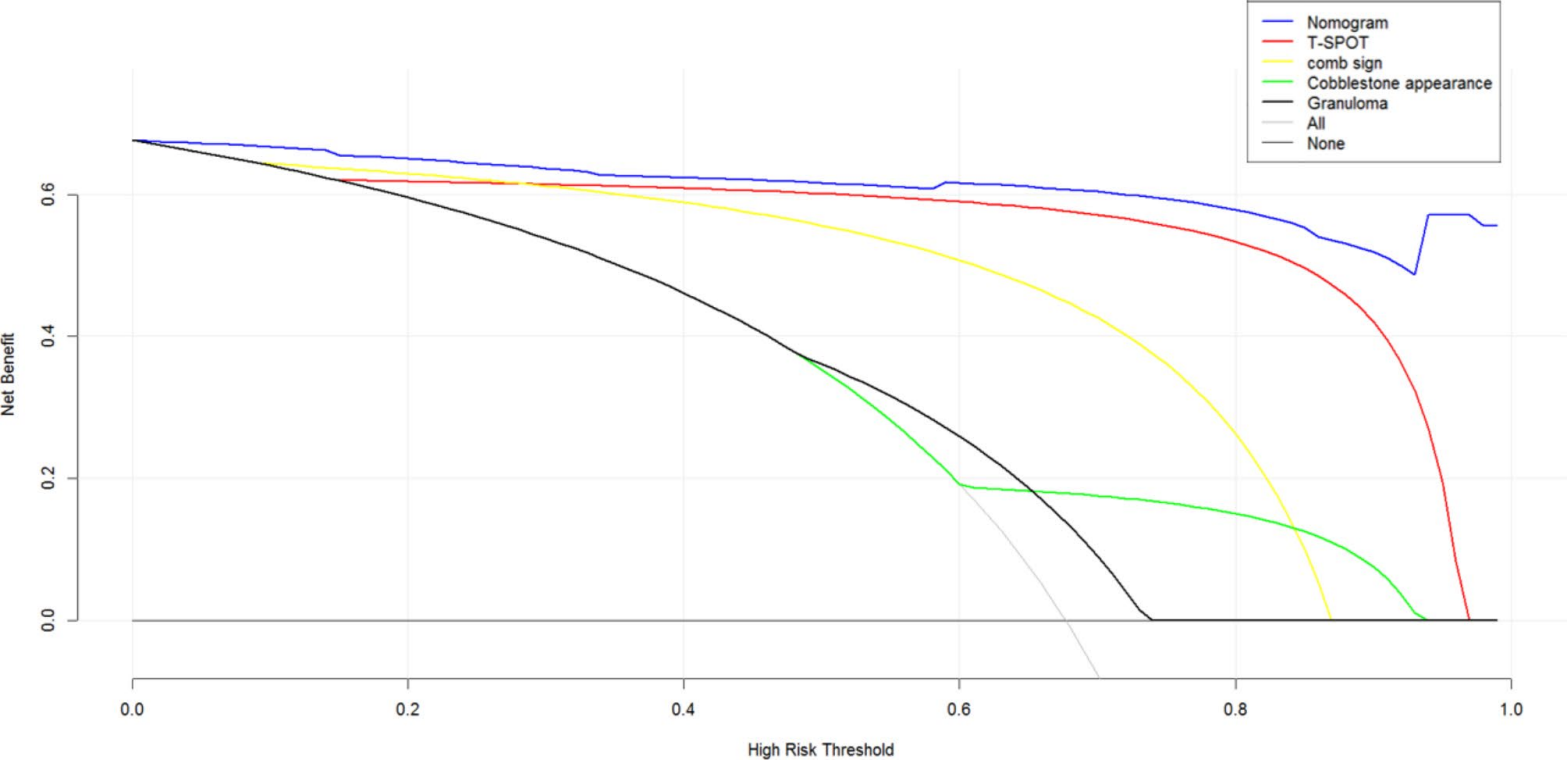
The decision curve analysis of the nomogram prediction model and independent predictive factors

The net benefits between the nomogram verses each factor in predicting the probability of CD were assessed by the decision curve analysis for their clinical usefulness. In this analysis, nomogram provided a higher net benefit than all other factors across all ranges of the threshold probability, suggesting that clinical intervention guided by this nomogram provided a greater net benefit, this means that the nomogram prediction model could effectively guide clinical practice (Fig. 4).

**Fig. 5:**
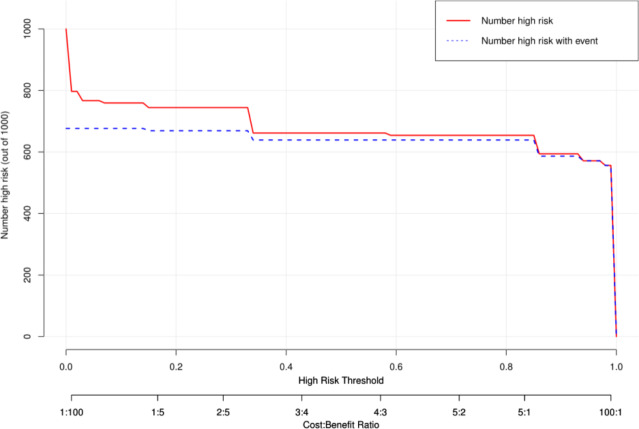
The clinical impact curve of the nomogram prediction model

On the basis of the DCA, the clinical impact curve was generated to analyze the number of patients classified as high risk by this nomogram and the number of true positive patients under each risk threshold. As shown in Fig. 5, the red solid curve represented the number of patients classified as CD by this nomogram under each risk threshold of 1,000 patients, and the blue dashed curve showed the number of true CD patients under each risk threshold. As the risk threshold decreased, the difference between the total number of patients predicted as Crohn’s disease by the model and the number of patients with true Crohn’s disease gradually expanded, implying a gradual increase in false positive rates and leading to an increase in the number of patients receiving unnecessary treatment. Therefore, to strike a balance between a higher net benefit rate and a lower false positive rate, we combined the DCA with the clinical impact curve. The alignment showed that when the risk of CD threshold is set at 0.90, it provides the higher clinical benefit and the lower false-positive rate to the entire included population.

## Discussion

CD and ITB are both chronic granulomatous diseases of the intestine that share similar clinical manifestations, endoscopic findings, CTE and histological features [1, 2]. In China, where tuberculosis is prevalent and the incidence of CD is increasing, it is important to accurately identify these two diseases in a timely and accurate manner. It is very important to make a correct differential diagnosis and take reasonable treatment measures in the early stage because of the distinct natural history and prognosis of these two disease. Thus, there is an urgent need to develop a better diagnostic tool to distinguish CD from ITB. In this research, we screened demographics, clinical manifestations, laboratory examination, endoscopic findings, CTE and pathological features in detail and searched for significant predictors for differentiating CD from ITB by univariate and multivariate logistic regression analyses. We found some clinical manifestations, laboratory examination, endoscopic findings, CTE and pathological features are helpful to distinguish CD from ITB. Among them, T-SPOT positive, cobblestone appearance, comb sign and granuloma are the most important features to distinguish CD from ITB. Cobblestone appearance and comb sign are the independent predictors of CD, while T-SPOT positive and granuloma are the independent predictors of ITB. On this basis, a nomogram prediction model for distinguishing CD from ITB was developed and assessment, which has high discrimination, calibration and clinical efficiency. It can be used as an accurate and convenient diagnostic tool to distinguish Crohn’s disease from intestinal tuberculosis, facilitating clinical decision-making.

The principal clinical manifestations of CD and ITB are abdominal pain, abdominal mass, and weight loss, accounting for more than 70%. However, the difference was not statistically significant, indicating that these main clinical manifestations lacked specificity in differential diagnosis. On univariate analysis of variables, diarrhea and perianal disease were more common in patients with CD, whereas ascites and pulmonary tuberculosis were suggestive of ITB, which is consistent with previous reports [1, 2, 17]. In addition, it is reported that the T-SPOT has superior sensitivity and specificity in the diagnosis of ITB compared with tuberculin skin test because it is not affected by previous Bacille de Calmette Guerin (BCG) vaccination and most nontuberculous mycobacteria infections [10, 18]. Our results showed that the sensitivity and specificity of T-SPOT are 92.2% and 93.0% in the differential diagnosis of CD and ITB, suggesting a valuable laboratory examination based on its high sensitivity and specificity, which is similar to previous reports [19].

Endoscopy plays an important role in the differential diagnosis of CD and ITB. The endoscopic features favoring CD included longitudinal ulcer, skip lesions and cobble stone appearance, whereas transverse (ring-shaped) ulcers favored the diagnosis of ITB in our study, as previously reported [2, 10, 20]. Our study also illustrated that intestinal ulcers are more common in the right colon in both Crohn’s disease and intestinal tuberculosis, especially in the ileocecum. One explanation is that the ileocecal region is commonly involved secondary to the high concentration of lymphoid aggregates in this area and prolonged contact between the bacilli and ileocecal mucosa [21]. Involvement in the terminal ileum, transverse colon, descending colon, sigmoid colon and the rectum was significantly more frequent in patients with CD than ITB, which is also consistent with previous studies [12, 22].

Computed tomographic enterography (CTE) are the preferred imaging modalities for evaluating and differentiating between patients with CD and ITB [22]. In our study, we found that asymmetrical bowel wall thickening, skip lesion, segmental small-bowel lesions, target sign, the comb sign, mesentery fibrofatty proliferation and homogeneous enhancement of lymph nodes were significantly more common in Crohn’s disease patients, which could provide reference for CD and ITB differentiation, as previously reported [3, 23]. Inflammatory stimuli leads to the increasing of small blood vessels around the lesion, which is shown as “comb sign” on CTE in patients with CD. Park et al. pointed out that a positive comb sign was the most suggestive finding of CD [24]. Mao et al. found that segmental small bowel involvement and comb sign were independent predictors of CD. Combining CTE and colonoscopic findings increased the accuracy of diagnosing either CD or ITB [3]. Visceral fat is a component of mesenteric fat and mesenteric fatty proliferation is one of the hallmarks of CD, being recognized as early as 1932 by Burril B. Crohn [25]. Fat hypertrophy, fat wrapping, and creeping fat have been associated with active CD [22]. We also found that mesentery fibrofatty proliferation favored the diagnosis of CD in our study.

Pathological features are the key to distinguishing ITB from CD. The “gold standard” typical granulomas with caseous necrosis for the diagnosis of intestinal tuberculosis limits its value in clinical application due to the low positive rate. In our study, caseous necrosis was found in only 7.0% of ITB patients, which was lower than the reported positive rate about 11.1% [4]. Our data presented in this study demonstrated that compared with CD patients, granuloma was more common in ITB patients, and the difference was statistically significant, which could provide a reference for the differential diagnosis. Yu et al. also found that granuloma was more common in intestinal tuberculosis than in CD. Furthermore, night sweats, longitudinal ulcers and granulomas were the most important features to differentiate Crohn’s disease from intestinal tuberculosis on further multivariable logistic regression analysis [13]. Tubercular granulomas are usually large (> 200 μm), dense, confluent, located in submucosa, however granulomas in CD are usually small (microgranuloma), discrete, sparse and can be situated either in the mucosa or in the submucosa [1, 13, 17]. Surgical specimens may reveal the presence of fissure-like ulcers, which are more common in CD and may extend till serosa, whereas they are rare in ITB, and if present they usually do not extend beyond the submucosa. In our study, fissure-like ulcers were found in a small proportion of CD patients about 3.3%, which are the limitation in clinical practice.

Although many valuable parameters of clinical manifestations, endoscopic findings, CTE and histological features discussed above help to differentiate between CD and ITB, the value of using a single parameter in distinguishing these two disease is very limited in clinical practice due to low sensitivity or specificity. Thus, establishing diagnostic model with multiple selected valuable parameters may be expected to obtain a diagnostic method with high accuracy and clinical maneuverability. Lee et al. established a diagnostic model base on eight colonoscopy parameters and showed a positive predictive value for CD as 94.4%, a positive predictive value for ITB as 88.9%, and accuracy 95.5% [20]. Li et al. established a model based on clinical features and an endoscopic model in their study. Both these models had moderate sensitivity and specificity of approximately 80% in differentiating CD and ITB [21]. In another study from China, two models were established based on clinical and CTE features, which showed diagnostic accuracy of 91.0% and 95.7%, respectively [12]. All above models were established based on single or limited examination tools with quite different sensitivity, specificity and accuracy and no validation was performed.

In a study from India, Makharia et al. established a diagnostic model including blood in the stool, weight loss, histological focally enhanced colitis, and involvement of the sigmoid colon recruited parameters from clinical manifestation, endoscopic findings, and pathologic features with 83% sensitivity, 79.2% specificity, and 81.1% diagnostic accuracy [1]. Yu et al. recruited independent predictors for diagnosis of CD and ITB included night sweats, longitudinal ulcers, and granulomas as variables for the predictive model, which had good diagnostic accuracy with an AUC of 0.86 [13]. Jung et al. formulated a predictive model including age, sex, ring-shaped ulcers, suspicion of radiological pulmonary tuberculosis, longitudinal ulcers, diarrhea, and sigmoid colon involvement in a Korean population and showed a better performance, with a sensitivity of 95.9%, a specificity of 94.9%, and the AUC of 0.979 [2]. The above models reported the diagnostic yield of differential diagnosis with big variations. Besides, most of these models included only one or some examination tools and have not evaluated all the features, in particular lack of radiological features. Especially, the formulae used in these prediction models are complicated and difficult to be applied in clinical practice.

Recently, He et al. establish two models based on 7 differential variables: age, transverse ulcer, rectum involvement, skipped involvement of the small bowel, target sign, comb sign, and interferongamma release assays (for model 1) or purified protein derivative (for model 2), respectively [16]. Accordingly, two nomograms of the above two models were developed for clinical practical use respectively and the nomogram 1 with 92.4% specificity, 95.8% sensitivity, 94.7% accuracy for diagnosing CD, and the nomogram 2 with 90.9% specificity, 82.5% sensitivity, 82.1% accuracy for diagnosing CD, which can be conveniently used to identify some difficult CD or ITB cases, allowing for decision-making in a clinical setting. However, these models do not include pathological features, which has an important role in differentiating CD from ITB, and recruit feature (age) which may not be applicable to other populations. Furthermore, they do not evaluate the calibration and clinical usefulness of the models.

The present study comprehensively screened variables with statistical differences from demographics, clinical manifestations, laboratory examination, endoscopic findings, CTE and pathological features. Multivariate logistic regression analysis showed that T-SPOT positive, cobblestone appearance, comb sign and granuloma were significant predictors in differentiating CD and ITB. Base on the above multivariate analysis, a nomogram prediction model to distinguish CD from ITB was successfully established with the higher sensitivity, specificity, accuracy of 94.4%, 93.0%, 94.0%, respectively. The high C-index as well as the area under the curve showed that this prediction model can be widely and accurately used to distinguish CD from ITB. And the nomogram showed good internal calibration between the actual observation and the prediction in the derivation cohort. In addition, the DCA and clinical impact curves were employed to determine a clinical decision point that the patients could obtain the highest net benefit. Taken together, the present nomogram can be used as an accurate and convenient diagnostic tool to distinguish CD from ITB, facilitating clinical decision-making.

Our study has several strengths. First, this present study developed a diagnostic nomogram prediction model to differentiate between CD and ITB, with intuitive, easy-touse characteristics. Second, screening variables are more comprehensive, especially the inclusion of radiological and pathological features in our study. Third, this study employed for the first time the DCA and clinical impact curve to evaluate the clinical efficiency of diagnostic nomogram. However, there are some shortcomings in our study that this is a single-center study with a limited number of patients and we validated our model in the same data set due to the limited number of patients, so further studies with a larger sample size from multiple centers are needed to validate this predictive model. Besides, this model may not be applicable in other countries and regions, further research conducted among other populations is warranted to provide more evidence.

In conclusion, clinical manifestations, laboratory examination, endoscopic findings, CTE features and histological results are helpful to distinguish CD from ITB. T-SPOT positive, cobblestone appearance, comb sign and granuloma are the most important features to distinguish CD from ITB. On this basis, a nomogram prediction model for distinguishing CD from ITB was developed and assessed, which has high discrimination, calibration and clinical efficiency. It can be used as an accurate and convenient diagnostic tool to distinguish Crohn’s disease from intestinal tuberculosis, facilitating clinical decision-making.

## Electronic supplementary material

Below is the link to the electronic supplementary material.

Supplementary Material 1.
Supplementary Figure 1. The flow chart of this study.
Supplementary Figure 2 Typical endoscopic features in Crohn?s disease and and intestinal tuberculosis.
Supplementary Figure 3 Typical findings of computed tomographic enterography (CTE) in Crohn?s disease.
Supplementary Figure 4. Pathological features in Crohn’s disease and intestinal tuberculosis.
Supplementary Table 1. Comparison of general conditions of patients with CD and ITB.
Supplementary Table 2. Comparison of clinical manifestations and laboratory examination in patients with CD and ITB.
Supplementary Table 3. Comparison of endoscopic features and involved sites in patients with CD and ITB.
Supplementary Table 4. Comparison of imaging features in patients with CD and ITB.
Supplementary Table 5. Comparison of pathological features between CD and ITB.

## Data Availability

The datasets generated during the current study are not publicly available due to confidentiality of human subjects but are available from the corresponding author on reasonable request.
