# Supplementary material for: Differential diagnosis of Crohn’s disease and intestinal tuberculosis: development and assessment of a nomogram prediction model
Source: BMC Gastroenterol. 2022 Nov 16;22:461. doi: 10.1186/s12876-022-02519-z (PMC9670453; doi:10.1186/s12876-022-02519-z)
Supplement: Supplementary file 1 — Supplementary Material 1. Supplementary Figure 1. The flow chart of this study. Supplementary Figure 2 Typical endoscopic features in Crohn?s disease and and intestinal tuberculosis. Supplementary Figure 3 Typical findings of computed tomographic enterography (CTE) in Crohn?s disease. Supplementary Figure 4. Pathological features in Crohn’s disease and intestinal tuberculosis. Supplementary Table 1. Comparison of general conditions of patients with CD and ITB. Supplementary Table 2. Comparison of clinical manifestations and laboratory examination in patients with CD and ITB. Supplementary Table 3. Comparison of endoscopic features and involved sites in patients with CD and ITB. Supplementary Table 4. Comparison of imaging features in patients with CD and ITB. Supplementary Table 5. Comparison of pathological features between CD and ITB. [file 12876_2022_2519_MOESM1_ESM.docx]

**
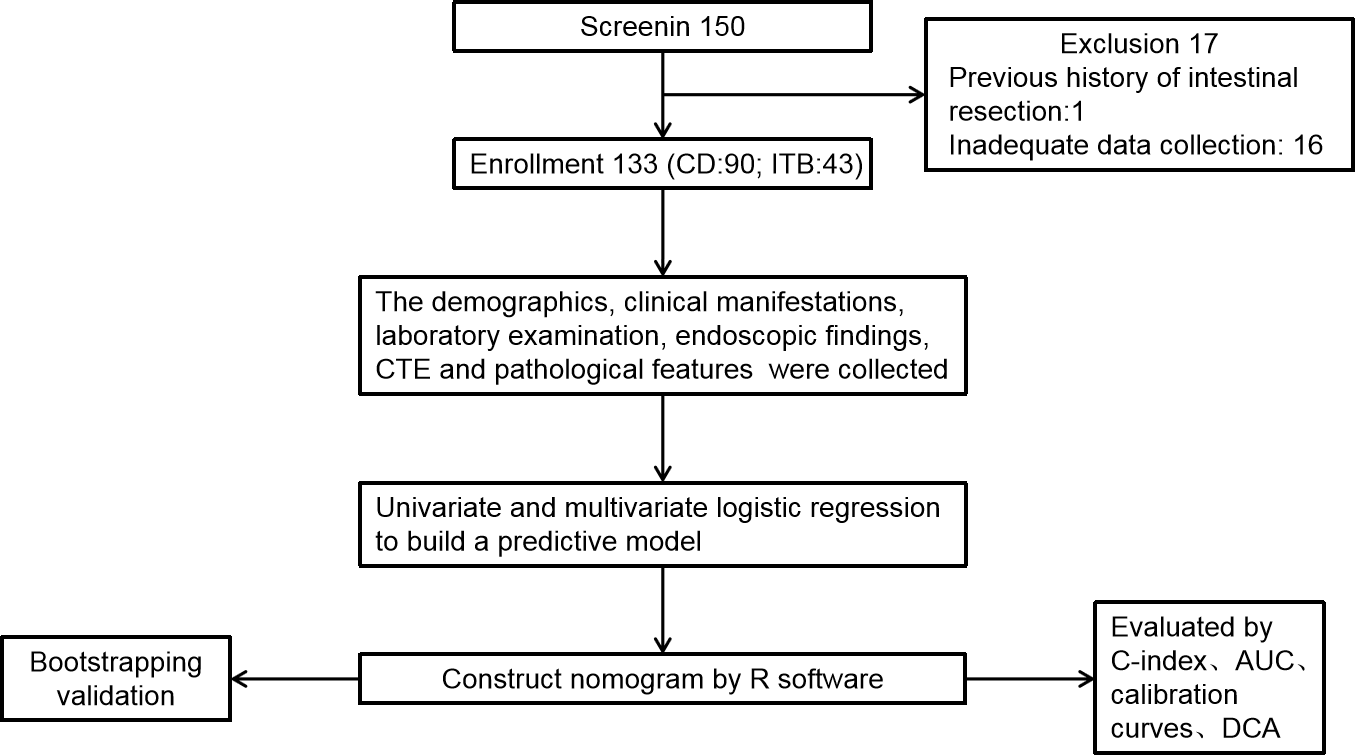
**

Supplementary Figure 1. The flow chart of this study


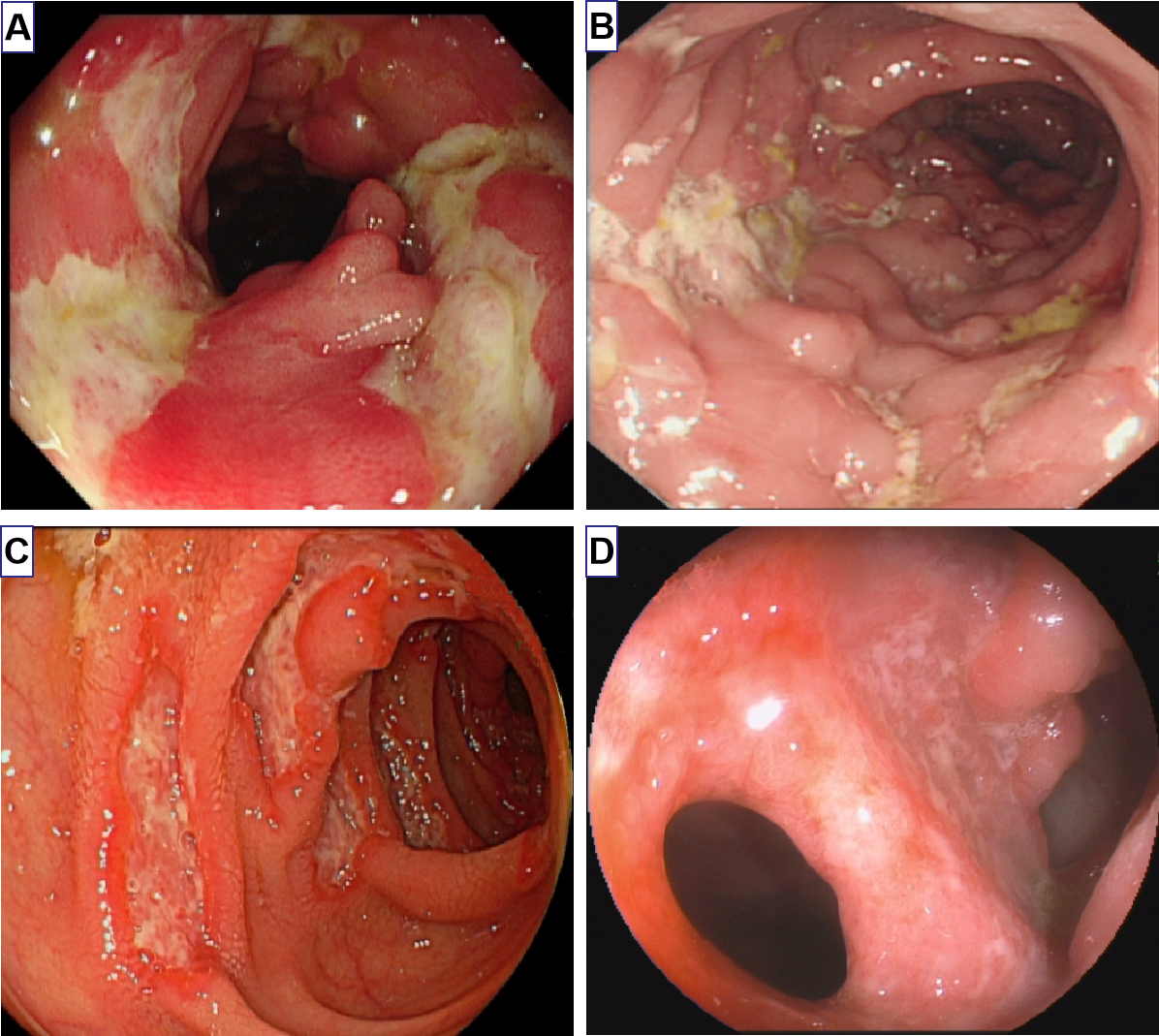


Supplementary Figure 2 Typical endoscopic features in Crohn’s disease and and intestinal tuberculosis

A:Longitudinal ulcer in a patient with CD; B:Cobblestone appearance in a patient with CD; C:Transverse ulcer in a patient with ITB; D:Patulous ileocecal valve in a patient with intestinal tuberculosis


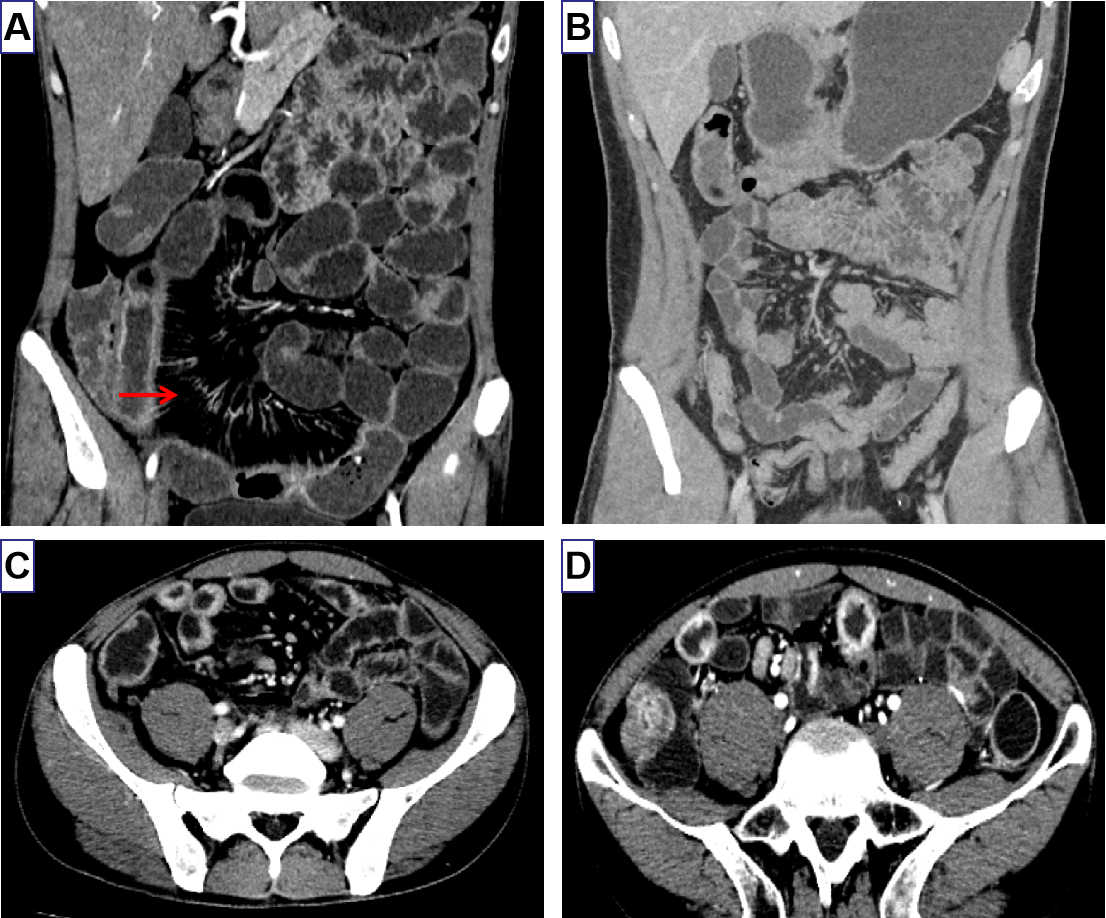


Supplementary Figure 3 Typical findings of computed tomographic enterography (CTE) in Crohn’s disease

A:Comb sign (red arrow) in CD; B:Mesenteric fibrofatty proliferation in CD; C:Asymmetrical wall thickening and segmental small-bowel involvement in CD; D:Mural stratification (arrow) and segmental small-bowel involvement in CD.


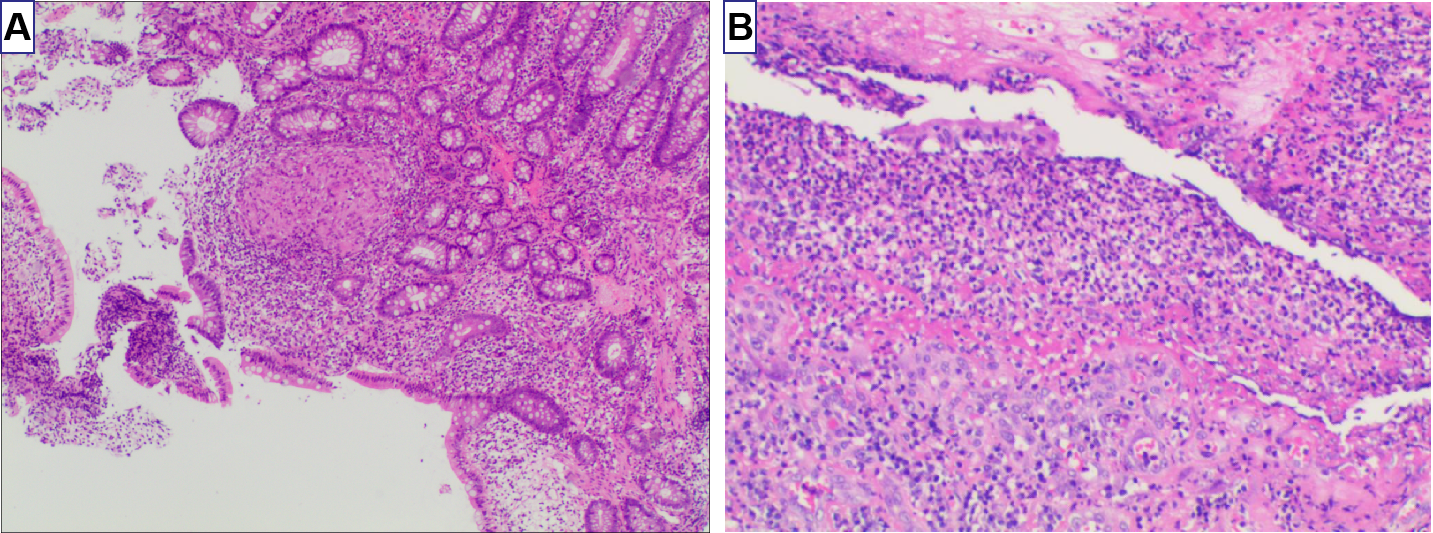


Supplementary Figure 4 Pathological features in Crohn's disease and intestinal tuberculosis

A: Granuloma was found in lamina propria in a patients with ITB; B: Fissure-like ulcer was found in a patients with CD.

SUPPLEMENTARY TABLE 1. Comparison of general conditions of patients with CD and ITB

| Variable | CD  (n=90) | ITB  (n=43) | *P* | OR（95% CI） |
| --- | --- | --- | --- | --- |
| Demographic features |  |  |  |  |
| Age, mean ± SD, y | 31.2±11.8 | 42.7±14.8 | 0.000 | - |
| Sex, male:female | 69:21 | 29:14 | 0.258 | - |
| Smoking history, n(%) | 17（18.9） | 15 (34.9) | 0.044 | 0.435(0.192-0.987) |

SUPPLEMENTARY TABLE 2. Comparison of clinical manifestations and laboratory examination in patients with CD and ITB

| Variable | CD  (n=90) | ITB  (n=43) | *P** | OR（95% CI） |
| --- | --- | --- | --- | --- |
| Clinical manifestations,n (%) |  |  |  |  |
| Abdominal pain | 73（81.1） | 36（83.7） | 0.714 | - |
| Diarrhea | 58（64.4） | 19（44.2） | 0.027 | 2.289(1.092-4.802) |
| Hematochezia | 24（26.7） | 7（16.3） | 0.185 | - |
| Constipation | 1（1.1） | 2（4.7） | 0.244 | - |
| Fever | 31（34.4） | 12（27.9） | 0.451 | - |
| Night sweats | 3（3.3） | 5（11.6） | 0.136 | - |
| Ascites | 0（0） | 4（9.3） | 0.010 | 0.907(0.824-0.998) |
| Weight loss | 66（73.3） | 31（72.1） | 0.880 | - |
| Abdominal mass | 6（6.7） | 6（14.0） | 0.294 | - |
| Intestinal obstruction | 6（6.7） | 7（16.3） | 0.152 | - |
| Perianal disease | 14（15.6） | 0（0） | 0.015 | 1.184(1.084-1.294) |
| Extraintestinal manifestations | 11（12.2） | 1（2.3） | 0.124 | - |
| Pulmonary tuberculosis | 0（0） | 26（60.5） | 0.000 | 0.395(0.273-0.572) |
| Laboratory examination,n (%) |  |  |  |  |
| T-SPOT positive | 7（7.8） | 40（93.0） | 0.000 | 0.006(0.002-0.026) |

*Pearson’s chi-square test or Fisher’s exact test.

SUPPLEMENTARY TABLE 3. Comparison of endoscopic features and involved sites in patients with CD and ITB

| Variable | CD  (n=90) | ITB  (n=43) | *P* | OR（95% CI） |
| --- | --- | --- | --- | --- |
| Endoscopic features, n (%) |  |  |  |  |
| Longitudinal ulcer | 35（38.9） | 1（2.3） | 0.000 | 26.727(3.517-203.804) |
| Transverse ulcers | 4（4.4） | 29（67.4） | 0.000 | 0.022(0.007-0.074) |
| Aphthous ulcers | 28（31.1） | 16（37.2） | 0.484 | - |
| Cobblestone appearance | 28（31.1） | 2（4.7） | 0.001 | 9.258(2.091-40.989) |
| Skip lesions | 57（63.3） | 6（14.0） | 0.000 | 10.652(4.065-27.909) |
| Patulous ileocecal valve | 7（7.8） | 6（14.0） | 0.418 | - |
| Intestinal stricture | 31（34.4） | 19（44.2） | 0.278 | - |
| Mucosal bridge | 5（5.6） | 5（11.6） | 0.373 | - |
| Scars or pseudopolyps | 15（16.7） | 13（30.2） | 0.073 | - |
| Site of involvement, n (%) |  |  |  |  |
| Terminal ileum | 65（72.2） | 22（51.2） | 0.017 | 2.482(1.166-5.281) |
| Ileocecal valve | 61（67.8） | 26（60.5） | 0.407 | - |
| Cecum | 68（75.6） | 33 (76.7） | 0.881 | - |
| [Ascending](E:/Program%20Files%20(x86)/Youdao/Dict/8.9.6.0/resultui/html/index.html#/javascript:;) [colon](E:/Program%20Files%20(x86)/Youdao/Dict/8.9.6.0/resultui/html/index.html#/javascript:;) | 47（52.2） | 21（48.8） | 0.715 | - |
| Transverse colon | 40（44.4） | 10（23.3） | 0.018 | 2.640(1.162-5.998) |
| Descending colon | 42（46.7） | 7（16.3） | 0.001 | 4.500(1.812-11.173) |
| Sigmoid colon | 45（50.0） | 6（14.0） | 0.000 | 6.167(2.370-16.048) |
| Rectum | 36（40.0） | 3（7.0） | 0.000 | 8.889(2.555-30.924) |

SUPPLEMENTARY TABLE 4. Comparison of imaging features in patients with CD and ITB

| Variable | CD  (n=90) | ITB  (n=43) | *P** | OR（95% CI） |
| --- | --- | --- | --- | --- |
| Morphology of involved bowel segments,n (%) |  |  |  |  |
| Concentric thickening | 46（51.1） | 26（60.5） | 0.311 | - |
| Asymmetrical thickening | 66（73.3） | 20（46.5） | 0.002 | 3.163(1.479-6.761) |
| Skip lesions | 43（47.8） | 9（20.9） | 0.003 | 3.456(1.487-8.031) |
| Segmental small-bowel Involvement | 65（72.2） | 18（41.9） | 0.001 | 3.611(1.686-7.733) |
| Type of enhancement pattern,n (%) |  |  |  |  |
| Target sign | 77（85.6） | 22（51.2） | 0.000 | 5.654(2.445-13.076) |
| Homogeneous enhancement | 24（26.7） | 21（48.8） | 0.011 | 0.381(0.178-0.814) |
| Mesenteric changes,n (%) |  |  |  |  |
| Comb sign | 87（96.7） | 13（30.2） | 0.000 | 66.923(17.838-251.071) |
| Mesenteric fibrofatty proliferation | 54（60.0） | 8（18.6） | 0.000 | 6.563(2.732-15.762) |
| Features of lymph nodes, n (%) |  |  |  |  |
| Central necrosis | 0（0） | 2（4.7） | 0.103 | - |
| Calcification | 0（0） | 1（2.3） | 0.323 | - |
| Greater than 1cm | 72（80.0） | 28（65.1） | 0.063 | - |
| Inhomogeneous enhancement | 2（2.2） | 3（7.0） | 0.328 | - |
| Homogeneous enhancement | 88（97.8） | 34（79.1） | 0.001 | 11.647(2.393-56.686) |
| Others,n (%) |  |  |  |  |
| Peritoneal thickening | 26（28.9） | 11（25.6） | 0.691 | - |
| Ascites | 13（14.4） | 8（18.6） | 0.538 | - |
| Fistula | 4（4.4） | 0（0） | 0.304 | - |
| Abscess | 2（2.2） | 0（0） | 1.000 | - |

*Pearson’s chi-square test or Fisher’s exact test.

SUPPLEMENTARY TABLE 5. Comparison of pathological features between CD and ITB

| Variable | CD  (n=90) | ITB  (n=43) | *P** | OR（95% CI） |
| --- | --- | --- | --- | --- |
| Chronic active inflammation | 90（100） | 43（100） | - | - |
| Abnormal crypt structure | 18（20） | 3（7.0） | 0.054 | - |
| Cryptitis and crypt abscess | 14（15.6） | 2（4.7） | 0.071 | - |
| Granuloma | 15（16.7） | 16（37.2） | 0.009 | 0.338（0.147-0.774） |
| Transmural inflammation | 2（2.2） | 0（0） | 1.000 | - |
| Fissure-like ulcers | 3（3.3） | 0（0） | 0.551 | - |
| Caseous necrosis | 0（0） | 3（7.0） | 0.032 | 0.930（0.857-1.010） |

*Pearson’s chi-square test or Fisher’s exact test.
